# Induction of autophagy by cystatin C: a potential mechanism for prevention of cerebral vasospasm after experimental subarachnoid hemorrhage

**DOI:** 10.1186/2047-783X-18-21

**Published:** 2013-07-01

**Authors:** Yizhi Liu, Hongfa Cai, Zhong Wang, Jianke Li, Kaiyong Wang, Zhengquan Yu, Gang Chen

**Affiliations:** 1 Department of Interventional Radiology, The First Affiliated Hospital of Soochow University, 188 Shizi Street, Suzhou 215006, Jiangsu Province, China; 2 Department of Neurosurgery, The First Affiliated Hospital of Soochow University, 188 Shizi Street, Suzhou 215006, Jiangsu Province, China

**Keywords:** Autophagy, Cerebral Vasospasm, Cystatin C, Subarachnoid Hemorrhage

## Abstract

**Background:**

Studies have demonstrated that autophagy pathways are activated in the brain after experimental subarachnoid hemorrhage (SAH) and this may play a protective role in early brain injury. However, the contribution of autophagy in the pathogenesis of cerebral vasospasm (CVS) following SAH, and whether up-regulated autophagy may contribute to aggravate or release CVS, remain unknown. Cystatin C (CysC) is a cysteine protease inhibitor that induces autophagy under conditions of neuronal challenge. This study investigated the expression of autophagy proteins in the walls of basilar arteries (BA), and the effects of CysC on CVS and autophagy pathways following experimental SAH in rats.

**Methods:**

All SAH animals were subjected to injection of 0.3 mL fresh arterial, non-heparinized blood into the cisterna magna. Fifty rats were assigned randomly to five groups: control group (n = 10), SAH group (n = 10), SAH + vehicle group (n = 10), SAH + low dose of CysC group (n = 10), and SAH + high dose of CysC group (n = 10). We measured proteins by western blot analysis, CVS by H&E staining method, morphological changes by electron microscopy, and recorded neuro-behavior scores.

**Results:**

Microtubule-associated protein light chain-3, an autophagosome biomarker, and beclin-1, a Bcl-2-interacting protein required for autophagy, were significantly increased in the BA wall 48 h after SAH. In the CysC-handled group, the degree of CVS, measured as the inner BA perimeter and BA wall thickness, was significantly ameliorated in comparison with vehicle-treated SAH rats. This effect paralleled the intensity of autophagy in the BA wall induced by CysC.

**Conclusions:**

These results suggest that the autophagy pathway is activated in the BA wall after SAH and CysC-induced autophagy may play a beneficial role in preventing SAH-induced CVS.

## Background

Cerebral vasospasm (CVS) is a frequent and devastating complication in patients with cisternal subarachnoid hemorrhage (SAH) and represents a significant cause of morbidity and mortality in neurosurgical patients [1]. Despite promising therapeutic approaches, such as triple-H therapy, calcium channel blockades, sodium nitroprusside, and endothelin-receptor antagonists, successful treatment after SAH remains inadequate and the underlying pathogenic mechanisms of CVS remain unidentified.

Autophagy is a cellular process of “self-digestion”. When cells encounter stress conditions, such as nutrient limitation, heat, oxidative stress, and/or the accumulation of damaged or excess organelles and abnormal cellular components, autophagy is induced as a degradative pathway. The elimination of potentially toxic components coupled with the recycling of nutrients aids in cell survival [2]. Autophagy pathway activation may play an important role in several central nervous system (CNS) diseases, such as cerebral ischemia [3], hypoxia-ischemia induced brain injury [4], traumatic brain injury [5], intracerebral hemorrhage [6], and SAH [7].

A previous report from our group [8] showed that autophagy was significantly increased in the cerebral cortex of rats and expression peaked at 24 h after induction of SAH. Early brain injury (EBI), seen as brain edema, blood–brain barrier impairment, cortical apoptosis, and clinical behavior changes, were significantly ameliorated by intracerebroventricular infusion of rapamycin (RAP, an autophagy activator). However, 3-methyladenine (an autophagy inhibitor) decreased expression of light chain-3 (LC3) and beclin-1, and aggravated the EBI, suggesting that the autophagy pathway may play a beneficial role in EBI development after SAH. Nevertheless, a literature review produced no studies that investigate the potential contribution of autophagy to CVS following SAH. Previous reports suggested that autophagy may suppress inflammation, oxidant activity and apoptosis, which had been shown to play a vital role in arterial wall thickening and vasculature stiffening following SAH [7,8]. The aim of the current study was to evaluate the expression of the autophagy pathway in the basilar artery (BA) wall in an experimental rat model of SAH and determine the potential role of autophagy induced by CysC in the development of CVS.

## Methods

### Animals

The animal use and care protocols, including all operation procedures, were approved by the Animal Care and Use Committee of Soochow University and conformed to the Guide for the Care and Use of Laboratory Animals by the National Institute of Health, China. Fifty male Sprague–Dawley rats weighing from 300 to 350 g were purchased from the Animal Center of the Chinese Academy of Sciences (Shanghai, China). They were acclimated in a humidified room and maintained on a standard pellet diet at the Animal Center of Soochow University for at least 10 days. The temperature in both the feeding room and the operation room was maintained at 25°C.

### Subarachnoid hemorrhage (SAH) model

SAH was induced by the single-hemorrhage injection model in rats as previously described [9]. Briefly, after the animals were anesthetized with 4% chloral hydrate (400 mg/kg body weight) a small suboccipital incision was made, exposing the arch of the atlas, the occipital bone, and the atlanto-occipital membrane. The cisterna magna was tapped using a 27-gauge needle, and 0.3 mL of cerebral spinal fluid were gently aspirated. Non-heparinized freshly autologous blood (0.3 mL) from the femoral artery was then injected aseptically into the cisterna magna over a period of 2 min. Immediately after the injection of blood, the hole was sealed with glue to prevent fistula formation. The animals were tilted at a 30° angle for 30 min with their heads down, in a prone position, to permit pooling of blood around the BA. Afterwards, the rats were returned to their cages, the room temperature was kept at 23±1°C, and 20 mL of 0.9% NaCl was injected subcutaneously to prevent dehydration.

### Experimental design

Fifty rats were assigned randomly to five groups: control group (n = 10), SAH group (n = 10), SAH + vehicle group (n = 10), SAH + low dose of CysC group (n = 10), and SAH + high dose of CysC group (n = 10). CysC was dissolved in normal sodium, and the final concentrations were 2 μg/0.1 mL (low concentration) and 10 μg/0.1 mL (high concentration), respectively. A volume of 0.1 mL of the CysC dissolved in normal saline (NS) was administered directly into the cisterna magna 30 min before the blood injection as a means of prevention and treatment, while vehicle animals received an equal volume of NS only into the cisterna magna.

The rats were re-anesthetized and euthanized 48 h after blood injection by means of transthoracic cannulation of the left ventricle; they were perfused with 300 mL of phosphate-buffered saline solution under a pressure of 120 cmH_2_O. The BAs were immediately removed and 5 of 10 specimens in each group were placed in the fixative solution (a mixture of 4% paraformaldehyde and 2.5% glutaraldehyde in 0.1 M phosphate buffer, pH 7.4) for 24 h for histopathological examination and morphometric analysis, and another five specimens were frozen in liquid nitrogen for Western blot analysis.

### Morphometric measurements

The BA luminal perimeter and wall thickness for each specimen was measured using a digitized image analysis system with Image-pro Plus software. The specimens for light microscopy study were dehydrated in graded ethanol, embedded in paraffin, sectioned, and stained with hematoxylin and eosin. Light microscopic sections of arteries were projected as digitized video images. The inner perimeters of the vessels were measured by tracing the luminal surface of the intima. The thickness of the vessel wall was determined by taking four measurements of each artery that extended from the luminal surface of the intima to the outer limit of the media, to avoid inclusion of the adventitia. The four measurements were averaged.

### Western blotting analysis

The frozen brain samples were mechanically lysed in 20 mM Tris, pH 7.6, containing 0.2% sodium dodecyl sulfate (SDS), 1% Triton X-100, 1% deoxycholate, 1 mM phenylmethylsulphonyl fluoride, and 0.11 IU/mL aprotinin (all purchased from Sigma-Aldrich). Lysates were centrifuged at 12,000 ×g for 20 min at 4°C. The protein concentration was estimated by the Bradford method using the Nanjing Jiancheng protein assay kit (Nanjing Jiancheng Bioengineering Institute, Nanjing, China). The samples (60 μg per lane) were separated by 8% SDS polyacrylamide gel electrophoresis and electro-transferred onto a polyvinylidene-difluoride membrane (Bio-Rad Lab, Hercules, CA, USA). The membrane was blocked with 5% skimmed milk for 2 h at room temperature, incubated overnight at 4°C with primary antibodies directed against LC-3 and beclin-1 (Santa Cruz Biotechnology, Santa Cruz, CA, USA) at the dilutions of 1:200 and 1:150, respectively. Glyceraldehyde-3-phosphate dehydrogenase (diluted in 1:6,000, Sigma-Aldrich) was used as a loading control. After the membrane was washed six times, for 10 min each time, in PBS plus Tween 20 (PBST), it was incubated in the appropriate HRP-conjugated secondary antibody (diluted 1:400 in PBST) for 2 h. The blotted protein bands were visualized by enhanced chemiluminescence Western blot detection reagents (Amersham, Arlington Heights, IL, USA) and were exposed to X-ray film. Developed films were digitized using an Epson Perfection 2480 scanner (Seiko Corp, Nagano, Japan). Optical densities were obtained using Glyko Bandscan software (Glyko, Novato, CA, USA). The tissue of five animals in each group was used for Western blot analysis at 48 h after SAH.

### Neurologic scoring

Three behavioral activity examinations (Table 1) were performed at 48 h after SAH using the scoring system reported previously to record appetite, activity, and neurological deficits [10].

**Table 1:** Behavior and activity scores

**Category**	**Behavior**	**Score**
Appetite	Finished meal	0
	Left meal unfinished	1
	Scarcely ate	2
Activity	Walk and reach at least three corners of the cage	0
	Walk with some stimulations	1
	Almost always lying down	2
Deficits	No deficits	0
	Unstable walk	1
	Impossible to walk	2

### Transmission electron microscopy

The brain tissue adjacent to the clotted blood was analyzed in this experiment. Samples for electron microscopy were fixed in phosphate-buffered glutaraldehyde (2.5%) and osmium tetroxide (1%). Dehydration of the cortex was accomplished in acetone solutions at increasing concentrations. The tissue was embedded in an epoxy resin. Semi-thin (1 μm) sections through the sample were then made and stained with toluidine blue; 600 Å-thin sections were made from a selected area of tissue defined by the semi-thin section, and these were stained with lead citrate and uranyl acetate. Brain ultrastructure was observed under a transmission electron microscope (JEM-1200X).

### Statistical analysis

All values are expressed as means ± SEM. Statistical differences between the groups were compared using one-way ANOVA and Mann–Whitney U test. *P* values <0.05 were considered significant.

## Results

### General observations

**Figure 1:**
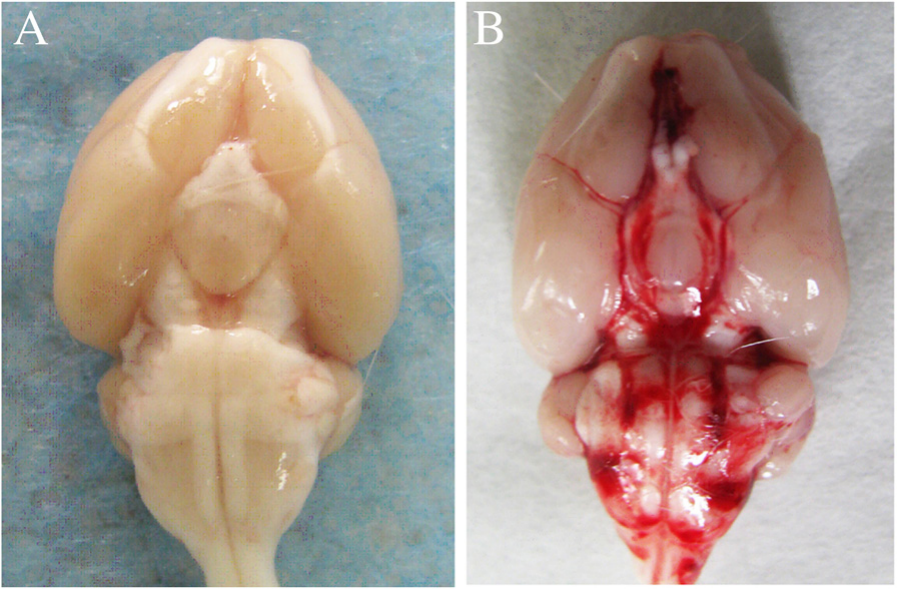
**Ventral view of typical brains in control group and SAH group. (A)** No blood clot was present in the control group. **(B)** Widespread distribution of blood was seen in the basal cisterns, the circle of Willis, and along the ventral brainstem in the SAH group.

There were no significant differences in body weight, temperature, or injected arterial blood gas data among the experimental groups (data not shown). After induction of SAH, all animals stopped breathing for about 15 s. The mortality rate of rats was 0% (0/10 rats) in the control group and 11% (5/45 rats) in the remaining groups. Widespread distribution of blood was seen in the basal cisterns, circle of Willis, and along the ventral brainstem 48 h after SAH. There were no blood clots in the control group (Figure 1).

### Morphometric vasospasm

**Figure 2:**
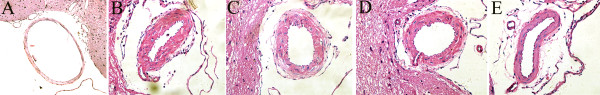
**Changes in the cross-sectional area of basilar arteries (BAs) in the experimental SAH model.** Representative images of cross-sectional areas of the BAs of the control group or rats subjected to SAH alone or SAH plus intracisternal injection with vehicle or CysC. **(A) **No corrugation and non-convoluted internal elastic lamina were observed in the control group; **(B)** Severe vasospasm could be detected in the SAH group; **(C)** Representative images showing luminal narrowing, increased wall thickness, and corrugation of the tunica intima in SAH + vehicle group. **(D-E)** The BA cross-sectional area was significantly increased in the SAH+ CysC group which was dose-dependent.

**Figure 3:**
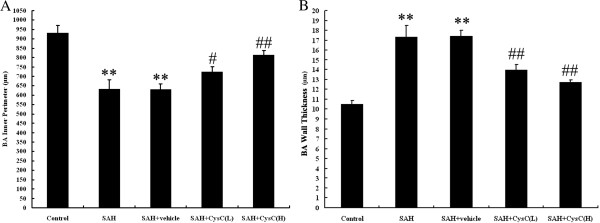
**The inner perimeter (A) and the wall thickness (B) of basal arteries (BAs).** Severe vasospasm was shown in SAH group and SAH + vehicle group. Decreased vasospasm was observed in rats treated with CysC. **P <0.01 compared with control group; #*P* <0.05 compared with SAH + vehicle group; ##*P* <0.01 compared with SAH + vehicle group.

The inner perimeter of BAs in the SAH group and vehicle group became smaller, and the BA wall thickness became thicker than in the control group (*P* <0.01). We observed moderate arterial narrowing and reduction of the intima in the above two groups. Compared with SAH and vehicle groups, the inner perimeter of the BA in the treatment group was expanded and thickness of BA walls decreased with a statistically significant difference (*P* <0.01), especially in the high dose group (compared with low concentration group, *P* <0.05) (Figures 2 and 3).

### Western blot analysis for detecting autophagy activation after SAH

**Figure 4:**
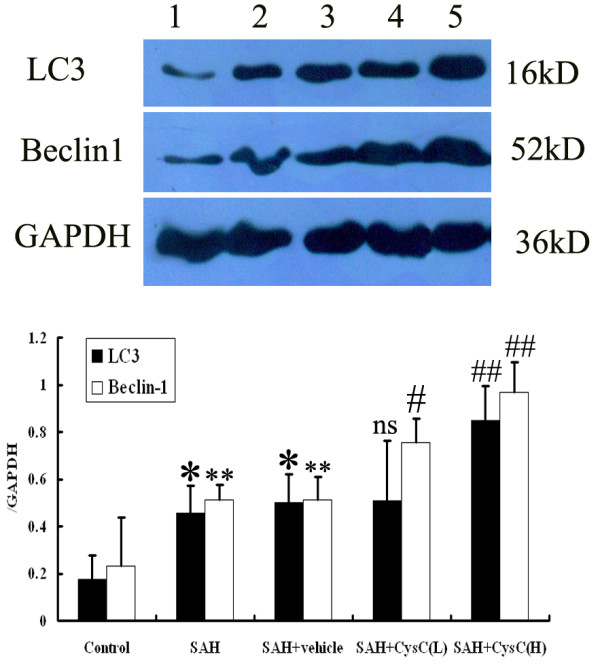
**Expressions of LC3 and beclin-1 in the BA walls in the control (n = 5, Lane 1), SAH (n = 5, Lane 2), SAH + vehicle (n = 5, Lane 3), SAH + low dose of CysC (n = 5, Lane 4), and SAH + high dose of CysC (n = 5, Lane 5) groups.** Upper: Representative autoradiograph showing protein expression following SAH by western blot. We detected LC3 at 16 kDa, beclin-1 at 52 kDa, and the loading control glyceraldehyde-3-phosphate dehydrogenase at 36 kD. Bottom: Quantitative analysis of the western blot results for the levels of LC3 and beclin-1. The expression of autophagy-related proteins was low in the control group. The expression of autophagy proteins was significantly increased in the SAH and SAH + vehicle experimental groups compared with controls (*P* <0.05). The increased expression was further markedly upregulated by CysC treatment (*P* <0.01). *P <0.05 compared with control group; **P <0.01 compared with control group; ns *P* >0.05 compared with SAH + vehicle group; #*P* <0.05 compared with SAH + vehicle group; ##*P* <0.01 compared with SAH + vehicle group.

Western blot analysis showed that the level of LC3 and beclin-1 in the BA wall was low in the control group. The expression of LC3 and beclin-1 was significantly increased at 48 h after blood injection in the SAH group and SAH + vehicle group (*P* <0.05). There was no statistically significant difference between the SAH group and SAH + vehicle group (*P* >0.05). After CysC injection, the level of LC3 and beclin-1 was markedly upregulated in animals of SAH + CysC group, especially in SAH + high concentration of CysC group (*P* <0.01) (Figure 4).

### Behavior and activity scores

As compared with the control group, clinical behavior function impairment caused by SAH was evident in SAH subjects (*P* <0.01). No significant difference was seen between the SAH group and SAH + vehicle group (*P* >0.05). CysC-treated rats showed better performance in this scale system than vehicle-treated rats 48 h after SAH, and the difference was statistically significant (*P* <0.01). There was no statistically significant difference between low and high concentration of CysC groups (*P* >0.05) (Table 2).

**Table 2:** Clinical behavior scales in each group

**Group**	**Mean score**
Control	0.4
SAH	2.5^*^
SAH + vehicle	2.6^ns^
SAH + low dose of CysC	1.7^**^
SAH + high dose of CysC	1.5^***^

^*^P <0.01, compared with control group; ^ns^*P* >0.05, compared with SAH group; ^**^P <0.01, compared with SAH + vehicle group; ^***^P <0.05, compared with SAH + low dose of CysC group.

### Transmission electron microscopy observations

**Figure 5:**
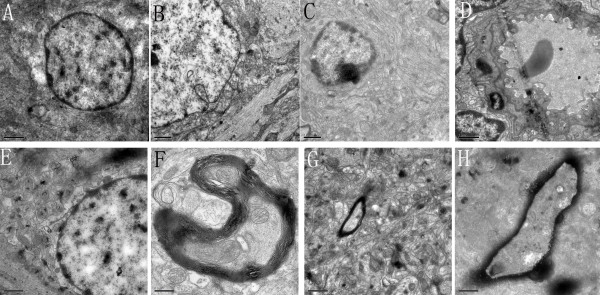
**Electron micrographs of the cortex at 48 h following sham operation (A), SAH induction (B and C), vehicle treatment (D), and CysC treatment (E, F, G, and H). (A)** In the control group, the glial cells were normal with integrated nuclear membrane. No swelling was found in endoplasmic reticulum and mitochondria. The electron density was normal in cytoplasm. **(B)** In the SAH group, the nuclear membrane was not integrated and the cytoplasm component entered the cell nucleus pushing the nuclear membrane. **(C)** In the SAH group, the nuclear membrane dissolved and shrank with nucleolar margination and chromatic agglutination in the glia cells. The staining was uneven with more endolysosome in the cytoplasm. **(D)** In the vehicle group, the endotheliocyte swelled in the capillary with apoptotic neurons and glial cells. **(E)** In the low dose CysC group, the nuclear membrane was more integrated than the SAH group with a little chromatic agglutination at the border of the nuclear membrane. In the cytoplasm, some of the mitochondria were swollen. **(F)** In the low dose CysC group, mild demyelination was found with the myelin sheath and mitochondria morphology was better than in the vehicle treated group. **(G-H)** In the high dose group, the myelin sheath was better than that in the SAH group surrounded with some stromal cells.

As shown in Figure 5, neurons and glial cells in the controls appeared healthy with normal endoplasmic reticulum, mitochondria, lysosomes, and nucleus. In contrast, diverse morphological changes were found in the cortex 48 h following SAH induction. Superficial neuroglial cells showed severe damage, such as cell harboring, multiple cytoplasmic vacuoles, cells completely lacking cytoplasmic contents, and shrunken nuclei with condensed chromatin. Numerous neurons displayed multiple vacuole-related structures containing electron-dense material or double membranous material. These pathological states were significantly ameliorated by CysC administration.

## Discussion

CVS is a common and potentially devastating complication in patients who have sustained SAH and is the most significant cause of morbidity and mortality in these patients [11]. In this present study, we investigated the role of CysC on CVS following SAH in rats, and explored possible mechanisms behind its actions. We made the following novel observations: 1) The pathological changes, including morphological changes, artery-narrowing, and thickening of BA wall, suggest that CVS occurs after SAH; 2) The level of expression of autophagy related proteins, LC-3, and Beclin-1, were low in the normal control group; 3) Autophagy was expressed in the BA wall during early stage after SAH in rats, suggesting that autophagy may participate in the pathological course of CVS; and 4) In CysC-handled group, the degree of CVS (inner perimeter of BA, BA wall thickness, and the clinical behavior function) was significantly ameliorated and this effect was paralleled with the intensity of autophagy in the BA wall induced by CysC. These findings suggest, for the first time, that SAH may induce vascular autophagy in the spasmed artery and might play a role in the pathogenesis of CVS. The therapeutic benefit of post-SAH CysC administration might be due to its salutary effect on modulating the autophagy signaling pathway.

CysC is an endogenous cysteine protease inhibitor, ubiquitously expressed and secreted in body fluids [12]. By inhibiting cysteine proteases such as cathepsins B, H, K, L, and S, it has a broad spectrum of biological roles in numerous cellular systems, with growth promoting activity, inflammation down-regulating function, and anti-viral and anti-bacterial properties [13]. It is involved in numerous and varied processes such as cancer, renal diseases, diabetes, and epilepsy, and neurodegenerative diseases such as Alzheimer’s disease.

Previous reports have shown that CysC plays a protective role in CNS diseases, such as Alzheimer's disease [14], focal brain ischemia [15], and progressive myoclonic epilepsy type 1, but did not elucidate the mechanism(s) of neuroprotection. Recently, Tizon et al. [14] demonstrated that CysC plays a protective role under conditions of neuronal challenge by inducing autophagy via mTOR inhibition. This neuroprotective function was prevented by inhibiting autophagy with beclin-1 siRNA or 3-methyladenine.

Accumulating evidence shows that the autophagy pathway plays an important role in the pathogenesis of different diseases in the CNS, such as cerebral ischemia [3], traumatic brain injury [5], experimental intracerebral hemorrhage [6], and hypoxia-ischemia brain injury [4]. In the SAH field, Lee et al. [7] demonstrated a significantly increased autophagic activity in the cortex in EBI after SAH. Our previous study [8] indicated that autophagy was significantly increased in the cortex of Sprague–Dawley rats and their expressions peaked 24 h after SAH. EBI such as brain edema, blood–brain barrier impairment, cortical apoptosis, and clinical behavior scale were significantly ameliorated by intracerebroventricular infusion of rapamycin (RAP, autophagy activator), while 3-methyladenine decreased expression of LC3 and beclin-1, and aggravated the EBI, suggesting that the autophagy pathway may play a beneficial role in EBI development after SAH. However, until now, no study has been found in the literature investigating the potential contribution of autophagy to CVS following SAH.

Autophagy also plays a housekeeping role in removing misfolded or aggregated proteins, clearing damaged organelles, such as mitochondria, endoplasmic reticulum and peroxisomes, and eliminating intracellular pathogens [16]. Failure of autophagy induces pleiotropic phenotypes leading to cell death, impaired differentiation, oxidative stress, toxic protein and organelle accumulation and persistence, tissue damage, inflammation, and mortality in mammals. This can lead to tissue dysfunction, inflammatory conditions, and cancer [17]. Previous publications suggested that autophagy can suppress inflammation [17,18], antioxidant activity [19-21], and anti-apoptosis [22,23] to maintain cellular homeostasis. Inflammation, oxidative stress, and apoptosis were considered to be a major component of SAH, and may contribute to the pathophysiology of both CVS and EBI [24]. It is indicated that the activation of autophagy may also have a beneficial role in the development of CVS following SAH. In this study, our data demonstrated that there is a significant increase of autophagy proteins in the BA wall 48 h following SAH induction, and the expression of autophagy was even higher after administration of CysC. In CysC-handled group, the degree of CVS, such as the inner perimeter of the BA and the BA wall thickness, was significantly ameliorated in comparison with vehicle-treated SAH rats, and this effect was paralleled with the intensity of autophagy in the BA wall induced by CysC.

## Conclusions

To the best of our knowledge, this is the first study to demonstrate the protective contribution of autophagy to CVS in the experimental SAH model, which suggests that the autophagy pathway may in fact play a significant role in CVS following SAH. Activation of autophagy induced by CysC resulted in attenuation of CVS in SAH models. Further studies evaluating the exact mechanism of autophagy pathway within CVS are warranted.

## Abbreviations

BA: Basilar arteries; CNS: Central nervous system; CVS: Cerebral vasospasm; CysC: Cystatin C; EBI: Early brain injury; LC3: Microtubule-associated protein light chain-3; RAP: Rapamycin; SAH: Subarachnoid hemorrhage.

## Competing interests

The authors declare that they have no competing interests.

## Authors’ contributions

YL, ZW, GC, and HC performed all experimental studies and data acquisition, and contributed to the study conception, design, analysis, and data interpretation. JL, KW, ZY, and GC collected samples, performed data analysis, and drafted the manuscript. All authors read and approved the final manuscript.
